# Evaluation on efficacy and safety of tyrosine kinase inhibitors plus radiotherapy in NSCLC patients with brain metastases

**DOI:** 10.18632/oncotarget.4264

**Published:** 2015-05-25

**Authors:** Shuimei Luo, Long Chen, Xiuping Chen, Xianhe Xie

**Affiliations:** ^1^ Department of Chemotherapy, The First Affiliated Hospital of Fujian Medical University, Fuzhou, Fujian, China; ^2^ Intensive Care Unit, The First Affiliated Hospital of Fujian Medical University, Fuzhou, Fujian, China; ^3^ Department of Oncology, Fuzhou Pulmonray Hospital, Fuzhou, Fujian, China

**Keywords:** non-small cell lung cancer, brain metastasis, tyrosine kinase inhibitor, radiotherapy, chemotherapy

## Abstract

**Objective:**

The study was designed to evaluate the efficacy and safety of tyrosine kinase inhibitors (TKIs) plus radiotherapy in patients with brain metastases (BM) of non-small cell lung cancer.

**Methods:**

Medline PubMed, Google Scholar, Web of Science, Oxford Journals Collection, clinical trials and current controlled trials were searched to identify relevant publications. After screening literature and undertaking quality assessment and data extraction, the meta-analysis was performed using RevMan5.3 software.

**Results:**

Eight controlled trials (980 participants) were included in the study. Compared with radiotherapy without TKIs (non-TKI-group), TKIs plus radiotherapy (TKI-group) had a significant benefit on objective response rate (ORR) (RR = 1.56, 95%CI [1.25,2.03]; *P* =0.0008), significantly prolonged the time to central nerves system progression (CNS-TTP) (HR =0.58, 95% CI [0.35, 0.96]; *P* =0.03) and median overall survival (MOS) (HR =0.68, 95% CI [0.47, 0.98]; *P* =0.04) of NSCLC patients with BM. There was no significant difference in overall severe adverse events (Grade≥3) (RR = 1.49, 95% CI [0.88,2.54]; *P* = 0.14) between two groups.

**Conclusion:**

This meta-analysis showed TKI-group produced superior response rate when compared with non-TKI-group. TKIs plus radiotherapy significantly prolong the CNS-TTP and MOS of patients without enhancing overall severe adverse events.

## INTRODUCTION

Worldwide, lung cancer ranks a top occurrence rate among malignant tumor with a rare 5-years survival rate (<15%) [1], of which non-small cell lung cancer (NSCLC) accounts for about 80%. Approximately 20-40% [2, 3] of NSCLCs develop brain mestastases (BM) with poor overall survival (OS) of only 3-6 months and severe neurological symptoms [4-6]. Current treatment options include surgical resection, whole brain radiation therapy (WBRT), stereotactic radiosurgery (SRS) alone or combined strategies. Radiotherapy remains the standard therapy for BM from NSCLC, however, long term results remain disappointing with a median survival time in the range of 2.4-4.8 months [7-9] due to the limitations of radiotherapy. Recent studies of radiotherapy in combination with conventional chemotherapeutics agents, such as platinum, nitrosourea, paclitaxel, temozolomide, suggest no significant improvement in OS compared with radiotherapy alone [10-15] owing to their low capacity of penetrating the brain-blood barrier (BBB). Thus, optimal treatment modalities are urgently needed for NSCLC patients with BM.

The epidermal growth factor receptor (EGFR) which expresses in a variety of human cancer cells, including ovarian, breast, colon, prostate and NSCLC [16, 17], is a transmembrane receptor protein identified primarily on cells of epithelial origin [18]. Autophosphorylation of its intracellar domain initiates a cascade of events leading to cell proliferation.

Fortunately, EGFR signal pathway can be blocked by small-molecule tyrosine kinase inhibitors (TKIs), including gefitinib and erlotinib, which targeting the EGFR to suppress cancer cell proliferation, invasion and metastases [19, 20]. Currently, TKIs have become increasingly important medications for advanced NSCLC treatment. Some studies showed favourable efficacy and safety in treating patients with BM [21-25] while other studies failed to confirm that [26, 27]. The role of TKIs plus radiotherapy for the treatment of BM patients is contraversial. Therefore, we have conducted a meta-analysis to assess the efficacy and safety of TKIs plus radiotherapy versus regimens with conventional chemotherapeautic agents plus radiotherapy or radiotherapy alone.

## RESULTS

### Selection of studies

**Figure 1:**
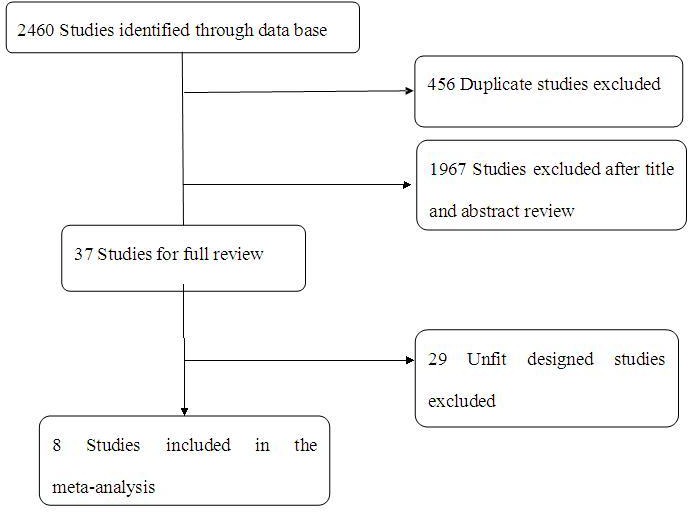
A flow chart on selection included of trials in the Meta-analysis

Totally, 2460 studies were screened which met our selection criteria after searching the relevant databases; 456 of these studies were excluded due to duplication. By verifying related terms in the titles and abstracts, 1967 irrelevant articles and another 29 unfit designed articles were excluded after the full text was analyzed. Finally, eight clinical control trials [21-28] were identified for the present meta-analysis. A flowchart depicting the study selection is shown in Figure 1.

### General characteristics of included studies

There were 980 patients with BM originating from NSCLC in the eight selected controlled trials, consisting of 374 patients with TKIs combined with radiotherapy, 376 patients with only radiotherapy, and 230 patients with conventional chemotherapy plus radiotherapy. These results are summarized in Table 1. Among these eight included studies, one was phase III clinical trials [27], three were phase II studies [21, 26, 28], and four studies didn't mention a trial phase [22-25]. Four of the studies involved in TKIs plus radiotherapy (TKI-group) versus radiotherapy alone (non-TKI-group) [21, 22, 24, 27], the others were TKIs combined with radiotherapy (TKI-group) versus conventional chemotherapy combined with radiotherapy (non-TKI-group) [23, 25, 26, 28]. Among all of the included studies, conventional chemotherapy drugs included placebo, temozolomide (TMZ), VMP, pemetrexed, gemcitabine, platinum, and other chemotherapy agents. Outcomes included ORR, MOS, CNS-TTP, and overall severe adverse event (grade≥3).

**Table 1:** Characteristics of trials included in the Meta-analysis

Study ID	country	Trial phase	N(T/C)	Male (T/C,%)	Ages (T/C,Years)	Interventions		outcomes					

						TKI-group	Non-TKI-group	ORR	OS	CNS-TTP	PFS	Adverse event (grade≥3)
Lee et al. 2014	Britain	II	40/40	37.5/52.5	61.3/62.2	erlotinib+WBRT	placebo+WBRT	N	Y	Y	N	Y
Zhuang et al. 2013	China	II	23/31	43/42	60/63	erlotinib+WBRT	WBRT	Y	Y	Y	Y	Y
Sperduto et al. 2013	America	III	41/44	N	61/64	erlotinib+WBRT/SRS	WBRT/SRS	N	Y	Y	N	Y
Fu et al. 2012	China	N	38/123	N	N	gefitinib+WBRT/SRS	WBRT/SRS	Y	N	N	N	Y
Pesce et al. 2011	Switzerland	II	16/43	56/63	57/63	gefitinib+WBRT	TMZ+WBRT	N	Y	N	N	Y
Wang et al. 2014	China	N	37/36	67.6/63.9	61/62	gefitinib+3D-CRT	VMP+3D-CRT	Y	Y	N	N	Y
Cai et al. 2014	China	N	104/178	59.6/66.3	65/65	TKI+WBRT/SRS/S	WBRT/SRS/S	N	Y	Y	N	N
Fan et al. 2013	China	N	75/111	57.3/73.0	57/57	TKI+WBRT/SRS/S	chemotherapy+WBRT/SRS/S	N	Y	N	N	N

**Abbreviation: N(T/C)**: number of patients(test group/control group); **ORR**: object response rate; **OS**: overall survival; **CNS-TTP**: time to central nerves system/neurological progression / neurological progression-free survival/progression-free survival of intracranial disease/local progression-free survival; **PFS**: progression-free survival; **N**: no mention in the paper; **Y**: have mentioned in the paper.

Data for all characteristics are summarized in Table 2. Sex, RPA(Radiation Therapy Oncology Group Recursive Partitioning Analysis), KPS (Karnofsky performance score), ECOG (Eastern Cooperative Oncology Group), No.of BM (number of brain metastases), extra-cranial metastases, histology were available for 6, 4, 4, 2, 6, 6, 6 of the 8 trials, respectively. Based on the available data, the histology of NSCLC were adenocarcinoma (61%).

**Table 2:** Characteristics of included patients

	TKI-group	Non-TKI-group
Sex		
Female	131(35%)	156(26%)
Male	164(44%)	283(47%)
unknown	79(21%)	167(27%)
RPA		
Grade 1	41(11%)	52(8%)
Grade 2	119(32%)	138(23%)
Grade 3	19(5%)	36(6%)
unknown	195(52%)	380(63%)
KPS		
<70	19(5%)	36(6%)
≥70	160(43%)	190(31%)
unknown	195(52%)	380(63%)
ECOG		
0-1	32(9%)	105(17%)
2	13(3%)	43(7%)
3-4	9(2%)	18(3%)
unknown	320(86%)	440(73%)
No.of BM		
≤3	203(54%)	265(44%)
>3	96(26%)	182(30%)
unknown	75(20%)	159(26%)
Extra-cranial metastases		
yes	173(46%)	266(44%)
no	126(34%)	181(30%)
unknown	75(20%)	159(26%)
Histology		
adenocarcinoma	228(61%)	323(53%)
squamous carcinoma	14(4%)	33(5%)
others	17(4%)	31(5%)
unknown	115(31%)	219(36%)

### Methodological quality

**Figure 2:**
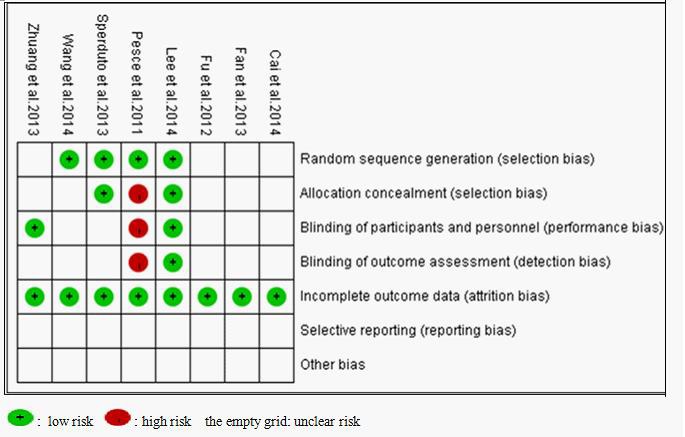
Bias risk and quality assessment of included studies

In accordance with the recommendations of the Cochrane Handbook for Systematic Reviews, we evaluated the eligible studies using the four aspects mentioned above. Four studies [23, 26, 27, 28] mentioned the use of random allocation, but only two of them discussed the methods [27, 28]. One study [21] performed or reported their allocation concealment and blinding methods. None of the trial reported follow-up information. All of the articles applied the intent-to-treat analysis. Seven of the eight eligible studies received B quality scores, only one received C quality scores, as shown in Figure 2.

### Local response rate

**Figure 3:**
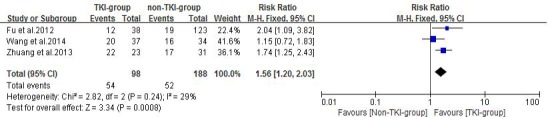
Objective response rate (ORR) of the study

Three of the included studies [21-23] reported response rate of treatment using TKIs plus radiotherapy versus conventional chemotherapy plus radiotherapy or radiotherapy alone. Zhuang et al. [21] reported intracranial tumor ORR in the erlotinib plus WBRT and WBRT alone groups were 95.65% and 54.84%, respectively. Fu et al. [22] reported intracranial tumor ORR in the gefitinib plus WBRT/SRS and WBRT/SRS alone groups were 31.6% and 15.4%, respectively. Wang et al. [23] reported intracranial tumor ORR were 54% and 47% in the gefitinib combined with 3D-CRT and VMP combined with 3D-CRT arms, respectively. A fixed effects model was used for the meta-analysis of these studies because heterogeneity did not exist (*P* = 0.24, *I^2^* = 29%). The results indicated that TKI-group produced superior response rates when compared with non-TKI-group (RR = 1.56, 95%CI [1.20, 2.03]; *P* =0.0008) as showed in Figure 3.

**Figure 4:**
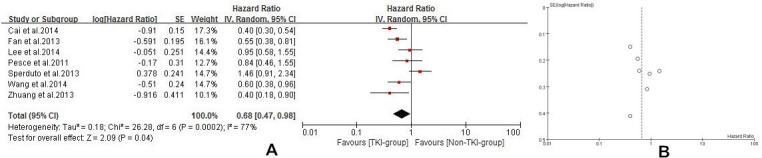
**A. Median overall survival (MOS) of the study B.** Funnel plot of MOS for included studies.

**Figure 5:**
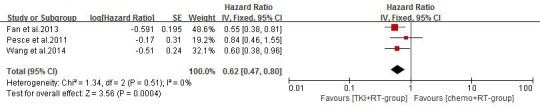
Median overall survival (MOS) of TKI plus radiotherapy *versus* chemotherapy plus radiotherapy

**Figure 6:**
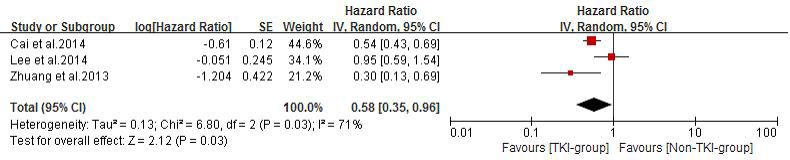
Time to central nerves system progression (CNS-TTP) of the study

Seven of the studies [21, 23-28] reported median overall survival (MOS) for both patient groups. Analysis using a random effects model based on the heterogeneity values (*P* = 0.0002, *I^2^* = 77%) of these studies suggested that in NSCLC patients diagnosed with BM, TKIs combined with radiotherapy significantly prolong MOS when compared with conventional chemotherapy combined with radiotherapy or radiotherapy alone (HR =0.68, 95% CI [0.47, 0.98]; *P* =0.04) (Figure 4A). The funnel plot indicated that there was no significant publication bias for included studies on MOS (Figure 4B). Subgroup analysis of TKI plus radiotherapy versus chemotherapy plus radiotherapy also demonstrated a desirable MOS in TKI-group (HR = 0.62, 95% CI [0.47, 0.80]; *P* = 0.0004) (Figure 5). Four studies [21, 24, 26, 27] reported CNS-TTP, and only three [21, 24, 26] with complete data were included in the analyzing using a random effects model based on the heterogeneity values (*P* = 0.03, *I^2^* = 71%), suggesting that TKIs plus radiotherapy significantly prolonged CNS-TTP (HR = 0.58, 95% CI [0.35, 0.96]; *P* = 0.03) (Figure 6).

### Adverse events

**Figure 7:**
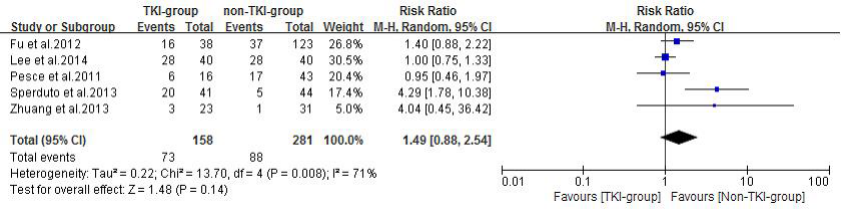
Overall severe adverse events of the study

Six enrolled studies had analyzed the treatment-related toxicity and adverse events, one of them (73 patients) [23] was excluded for not reporting the sufficient information of severe adverse events grading. A random effects model was used for the overall severe adverse events analysis of these studies based on the heterogeneity values (*P* = 0.008, *I^2^* = 71%). The results indicated that the incidence of overall severe adverse events did not differ between the TKI-group and non-TKI-group (RR = 1.49, 95% CI [0.88, 2.54]; *P* = 0.14) (Figure 7).

**Figure 8:**
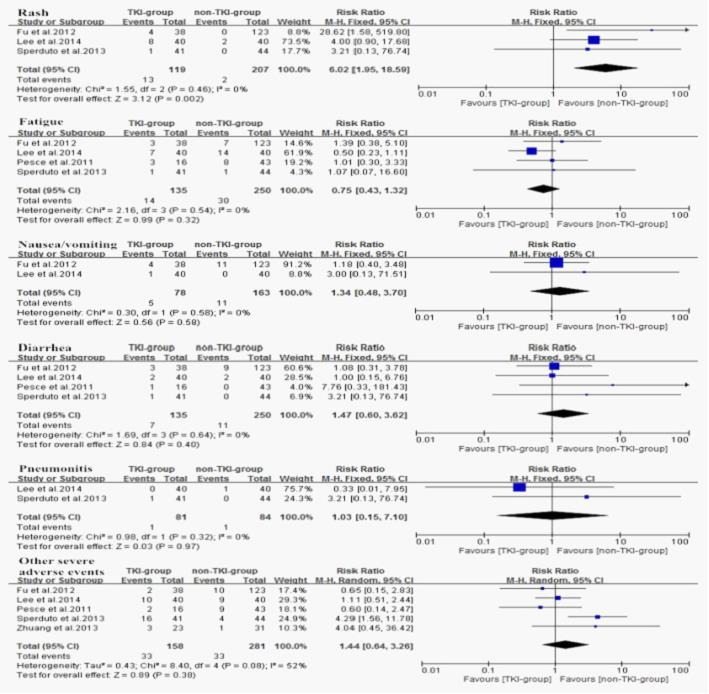
Subgroup analysis of severe adverse events

The most common adverse events of TKIs are rash, fatigue, nausea/vomiting, diarrhea which are largely mild and fairly tolerable, and pneumonitis rarely occurs. Thus, we performed a subgroup analysis for the severe adverse events as showed in (Figure 8). Regarding the fatigue, nausea/vomiting, diarrhea, pneumonitis, and other severe adverse events, no difference were observed with (RR = 0.75, 95%CI [0.43, 1.32]; *P* = 0.32), (R = 1.34, 95%CI [0.48, 3.70]; *P* = 0.58), (R = 1.47, 95%CI [0.60, 3.62]; *P* = 0.40), (R = 1.03, 95%CI [0.15, 7.10]; *P* = 0.97), (R = 1.44, 95%CI [0.64, 3.26]; *P* = 0.38). However, rashes were significantly more common in TKI-group (RR = 6.02, 95%CI [1.95, 18.59]; *P* = 0.002).

## DISCUSSION

Currently, local radiotherapy treatment remains the standard regimen of BM patients from NSCLC [32]. Several studies have certified that radiotherapy with chemotherapy benefits NSCLC patients with BM [33-35]. However, because penetration of most chemotherapeutic drugs into the central nervous system (CNS) is isolated primarily by the BBB [36], the treatment was unsatisfied at curing malignant BM lesions. Being small-molecule agents, TKIs possess great advantage to penetrate the BBB. The molecular pathways that mediate brain colonization and the alternative to traditional therapy in clinical investigations in BM from NSCLC have drawn widespread attention [37-41]. One pre-clinical study [42] showed that ^14^C radiolabeled gefitinib could be detected in the CNS of healthy mice after oral dose of gefitinib reached peak plasma concentrations, which suggested that gefitinib could penetrate the BBB, other studies [43-46] also showed that erlotinib appear good permeability through the BBB. Additionally, radiotherapy, immature tumor angiogenesis and edema might amplify the destruction of the BBB and enhanced TKIs uptake and elevated TKIs concentration in cerebrospinal fluid [47-53]. After penetrating into the BBB, TKIs exert their anti-cancer efficacy via following two mechanisms: one is competing with adenosine triphosphate (ATP), the other is to provide sufficient radiosensitizing and therapeutic level in the brain [54-58]. Recently, Togashi et al. [45] reported that CSF concentrations of erlotinib depend on its plasma concentration, and a high-dose administration of erlotinib could lead to its high CSF concentrations, therefore improved its efficacy, especially to refractory CNS metastases of NSCLC patients.

Compared with the conventional chemotherapeutic agents, TKIs appeared favorable effect in treating NSCLC patients with BM [23, 25, 28], which mainly resulted from the distinctive property of the drugs, including their small molecular structure and unique anti-tumor mechanism. Meanwhile, the most common adverse events of TKIs contain fatigue, rash, nausea, vomiting, diarrhea which in most cases are mild and tolerable, and interstitial pneumonia rarely appears. Our study demonstrated that there was no significant difference in overall severe adverse events between the TKI and non-TKI groups, except a tendency that non-TKI group had lower incidence of severe adverse events. This may due to the absence of chemotherapy in most of the non-TKI groups [21, 22, 24, 27].

In summary, the present study suggests TKIs combined with radiotherapy produced superior response rates, markedly prolong the CNS-TTP and MOS of NSCLC patients with BM without significantly enhancing overall severe adverse events. Moreover, it is possible to improve the efficacy through increasing the dosage of TKIs. TKIs plus radiotherapy probably open a promising avenue for treating NSCLC patients with brain metastases.

## MATERIALS AND METHODS

### Search strategy

Medline PubMed, Google Scholar, Web of Science, Oxford Journals Collection, randomized controlled trials (RCT) or clininal controlled trails were searched to identify relevant studies in the published literature. The search was performed on December 1, 2014, using both mesh and free text words. The following basic search terms were used: non-small cell lung cancer, brain metastasis, tyrosine kinase inhibitor, gefitinib, erlotinib, afatinib, radiotherapy, chemotherapy, WBRT, SRS, three-dimensional conformal radiotherapy (3D-CRT). The search was performed without any language limitations.

### Trial identification criteria

All projects which met the following criteria were eligible: (1) RCT or clinical controlled trails with voluntarily enrolled patients; (2) Patients had histologically or cytologically confirmed NSCLC and had been diagnosed with brain metastases using CT or MRI; (3) The trials were TKIs plus radiotherapy (WBRT/ SRS/ 3D - C RT alone or in combination) which were considered as TKI-group versus conventional chemotherapy plus radiotherapy or radiotherapy alone (both were considered as non-TKI-group). (4) Trials excluded patients with double or multiple primary cancer or presence of unstable systemic disease. (5) The analyses included objective response rate (ORR), OS, time to central nerves system/neurological progression (CNS-TTP) / neurological progression-free survival (nPFS)/local progression-free survival (LPFS) / progression-free survival of intracranial disease (PFSI) (all were considered as CNS-TTP in this study), severe adverse events (Grade≥3); (6) Response rate was determined using the Response Evaluation Criteria in Solid Tumors (RECIST1.0 or 1.1 version) [21-23]. Complete remission (CR) was defined as disappearance of all target lesions, any pathological lymph nodes (whether target or non-target) must have reduction in short axis to <10 mm. Partial response (PR) was defined as at least a 30% decrease in the sum of diameters of target lesions, taking as reference the baseline sum diameters. Progressive disease (PD) was defined as at least a 20% increase in the sum of diameters of target lesions, taking as reference the smallest sum on study (this includes the baseline sum if that is the smallest on study), in addition to the relative increase of 20%, the sum must also demonstrate an absolute increase of at least 5 mm. (Note: the appearance of one or more new lesions is also considered progression). Stable disease (SD) was defined as neither sufficient shrinkage to qualify for PR nor sufficient increase to qualify for PD, taking as reference the smallest sum diameters while on study. (7) Adverse events was evaluated according to the National Cancer Institute Common Terminology Criteria for Adverse Events (version 3.0) [22, 26, 27, 28].

### Study selection

The eligibility assessment was first performed by screening titles and abstracts and subsequently reviewing the full text of articles. The selection of all studies was executed independently, according to the inclusion criteria, by two reviewers [Shuimei Luo and Xiuping Chen]. Disagreement on whether an article should be included was resolved using a third reviewer [Xianhe Xie].

### Data extraction

Two authors [Shuimei Luo and Long Chen] independently extracted data from all the eligible studies. When the extracted data were not uniform, consultation was needed to make a final determination [Xianhe Xie]. All of the studies included in the analysis contain the following data: first author's name, published year, type of study, trial phase, country of origin study, percentage of male, performance status, number of patients, median ages, interventions and outcomes.

### Quality assessment

All of the selected studies were evaluated by two reviewers [Long Chen and Xianhe Xie] according to The Cochrane Handbook for Systematic Reviews of intervention (Version 5.1.0), based on the following criteria: (1) Random sequence generation; (2) Allocation concealment; (3) Blinding of participants and personnel; (4) Blinding of outcome assessment; (5) Incomplete outcome data; (6) Selective reporting; (7) Other bias. Each trial for bias based on the criteria listed above was marked as ‘low risk’, ‘high risk’ or ‘unclear risk’. Trials were judged as low risk of bias (i.e. A rating) when all criteria were assessed as low risk; Trials were judged as moderate risk of bias (i.e. B rating) or high risk of bias (i.e. C rating) when one or more criteria were assessed as unclear risk or high risk, respectively.

### Statistical analysis

Statistical analyses were performed using RevMan5.3. Chi-square and I-square tests were used to test the heterogeneity of different studies [29]; no heterogeneity was considered to exist when *P* > 0.1 and *I^2^* < 50%, a fixed-effect model was applied to pool the study results. Significant heterogeneity was found if *P* < 0.1 and *I^2^* > 50%, and a random-effects statistical model was used [30]. ORR, severe adverse events (Grade≥3) were analyzed using dichotomous variables. OS, CNS-TTP were calculated using effect variables. Hazard ratios (HRs) with 95% confidence intervals (CIs) were extracted from papers or from the survival curves when HRs were not available using the methods described by Zhou et al. [31] for CNS-TTP and OS. The event and total number of patients from TKI-group and non-TKI-group in the papers for object response rates (ORR) and severe adverse events, event-based relative risks (RR) with 95% CI were determined for ORR and severe adverse events extracted from 62.5% of the trials [21, 22, 26-28]. Publication bias was identified via funnel plot.
